# Gray matter volume alterations associated with suicidal ideation and suicide attempts in patients with mood disorders

**DOI:** 10.1186/s12991-020-00318-y

**Published:** 2020-12-10

**Authors:** Pengshuo Wang, Ran Zhang, Xiaowei Jiang, Shengnan Wei, Fei Wang, Yanqing Tang

**Affiliations:** 1 Department of Psychiatry, The First Affiliated Hospital of China Medical University, 155 Nanjing North Street, Heping District, Shenyang, 110001 Liaoning People’s Republic of China; 2 Brain Function Research Section, The First Affiliated Hospital of China Medical University, Shenyang, 110001 Liaoning People’s Republic of China; 3 Department of Gerontology, The First Affiliated Hospital of China Medical University, Shenyang, 110001 Liaoning People’s Republic of China; 4 Department of Radiology, The First Affiliated Hospital of China Medical University, Shenyang, 110001 Liaoning People’s Republic of China

**Keywords:** Mood disorders, Gray matter volume, Suicidal behavior, Suicide attempted, Suicidal ideation

## Abstract

**Background:**

Mood disorders are severe mental disorders related to increased suicidal behavior. Finding neural features for suicidal behavior, including suicide attempts (SAs) and suicidal ideation (SI), in mood disorders may be helpful in preventing suicidal behavior.

**Methods:**

Subjects consisted of 70 patients with mood disorders and suicidal behavior, 128 patients with mood disorders without suicidal behavior (mood disorders control, MC), and 145 health control (HC) individuals. All participants underwent structural magnetic resonance imaging (MRI). We used voxel-based morphometry (VBM) techniques to examine gray matter volumes (GMVs).

**Results:**

Significant differences were found in GMVs of the left and right middle frontal gyrus among the patients with mood disorders and suicidal behavior, MC, and HC. Post hoc comparisons showed significant differences in the GMVs of the above regions across all three groups (*P* < 0.01): HC > MC > mood disorders with suicidal behavior. However, there were no significant differences in the GMVs of the left and right middle frontal gyrus between the mood disorders with SI and mood disorders with SAs groups.

**Conclusions:**

These findings provide evidence that abnormal regional GMV in the middle frontal gyrus is associated with suicidal behavior in mood disorders. Further investigation is warranted to determine whether the GMV alterations in mood disorders with SI are different from these in mood disorders with SAs.

## Introduction

Suicide is a worldwide phenomenon and a serious social health problem. According to the World Health Organization, about 800,000 people die by suicide each year [1]. Suicidal ideation (SI) and suicide attempts (SAs) are strongly predictive of suicide death [2]. All over the world, lifetime prevalence rates are approximately 9.2% for SI and 2.7% for SA [3]. As typical mood disorders, previous studies have supported that major depressive disorder (MDD) and bipolar disorder (BD) have been widely reported to be associated with suicidal behavior [4–6]. For example, MDD is frequently associated with SAs and a large proportion of depressed individuals show SI [7]. In addition, 14–59% of patients with BD have SI and 25–56% present with at least one SA during their lifetime [8]. One study found that in mood disorders alexithymia may be considered risk factors for suicide [9]. Therefore, understanding the suicide risk factors of suicidal ideation and suicide attempts in patients with mood disorders is important and necessary for suicide prevention strategies.

Previous structural magnetic resonance imaging (MRI) studies have shown that abnormalities in gray matter volumes (GMVs) were associated with suicidal behavior in mood disorders. For example, gray matter volume reductions in the left and right dorsolateral prefrontal cortex (DLPFC) and right ventral lateral prefrontal cortex (VLPFC) were detected in patients with MDD and SI, as compared with those in patients with MDD without SI and HC groups [10]. The GMVs in the left limbic cingulated gyrus, the left angular gyrus, right cerebellum and right superior temporal gyrus, bilateral inferior temporal and superior temporal cortices, left superior parietal, thalamus and supramarginal regions, right insula, superior frontal and rostral middle frontal regions, DLPFC, anterior cingulate cortex, and the putamen and occipital cortex showed a significant decrease in the MDD with SAs group, as compared with those in the MDD with non-SAs group [11–16]. However, a previous study showed that GMVs in the DLPFC and orbitofrontal cortex (OFC) in patients with MDD with higher-lethality SAs were larger than those in patients with MDD with lower-lethality SAs and non-SAs [17]. For BD patients, the GMVs in the OFC, hippocampus, and cerebellum were reduced in the suicide attempters group when compared with the non-suicide attempters group [18, 19]. Prefrontal cortex gray matter volume was lower in patients with than without SAs in patients with BD and past psychiatric hospitalization, and prefrontal cortex gray matter volume was higher in patients with than without SAs in those BD cases without hospitalization [20]. Dante et al. showed that compared to the participants who had not had SAs, patients with BD who had experienced SAs exhibited a significantly increased gray matter volume in the right rostral anterior cingulate cortex [21]. Above neuroimaging evidence demonstrated abnormalities in the GMVs of patients with mood disorders and SI or SAs, which revealed its importance in terms of GMVs potentially being related to mood disorders with suicidal behavior. However, further understanding of the structural alterations of GMV in mood disorder patients with SI and SAs is limited but is needed to prevent suicide.

Previous studies on mood disorders with suicidal behavior have been conducted on a single sample, mainly in terms of SI or SAs. In the present study, we examined whole brain GMVs among patients with mood disorders and suicidal behavior (patients with suicidal behavior were divided into patients with SI and SAs), mood disorder patient controls, and healthy controls, using voxel-based analysis (VBA). We hypothesized that: (1) there would be alterations in the GMVs of mood disorder patients with suicidal behavior as compared with the BD patient controls; (2) there would be commonalities or differences in the GMVs between mood disorder patients with suicidal ideation and suicide attempts.

## Materials and methods

### Participants

All patients were from the Department of Psychiatry, Frist Affiliated Hospital of China Medical University, and the inpatient department of the Mental Health Center of Shenyang. HCs were recruited by an advertisement in the community. All participants had to be confirmed by two trained psychiatrists using the Structured Clinical Interview for DSM-IV Axis I Disorders (SCID-I). Patients with mood disorders were identified in accordance with the DSM-IV diagnostic criteria for MDD or BD, respectively, and did not meet the criteria for any other Axis I disorder. HC subjects had to have not had any current or lifetime Axis I Disorders, nor could they have had any first-degree relatives with a history of an Axis I disorder. All of the participants were assessed using the Hamilton Depression Rating Scale (HAMD) for symptoms of depression [22], and the Young Mania Rating Scale (YMRS) for manic symptoms [23]. Exclusion criteria for the study were as follows: (1) must not have had a history of major physical disorders, particularly those that may be associated with brain tissue changes, such as hypertension, diabetes, or metastatic disease; (2) must not have had unstable diseases such as heavy asthma; neurological abnormalities, including major head trauma (loss of consciousness lasting more than 5 min), epilepsy, cerebrovascular disease, brain tumors, or neurodegenerative diseases; somatic diseases that may cause mood disorders, such as multiple sclerosis, thyroid disease, etc.; (3) no MRI contraindications; and (4) no lifetime or current substance dependence or abuse. All participants signed informed consent as approved by the Ethics Committee of China Medical University.

The study included 343 subjects aged 15–49 years divided into three groups: 70 patients with mood disorder and suicidal behavior (mean age: 27.529 ± 9.618 years; 55 females), 128 mood disorder patients without suicidal behavior (mood disorders control, MC) (mean age: 27.141 ± 8.318 years; 93 females), and 145 health control (HC) individuals (mean age: 27.833 ± 9.498 years; 96 females). Then, the patients with mood disorders and suicidal behavior were divided into two groups: Mood disorders with SI (SI defined as thoughts of engaging in behavior intended to end one’s life, assessed by the Beck 19-item Scale for Suicide Ideation [24]) and mood disorders with SAs (i.e., at least one attempt defined as a self-destructive act with some degree of intent to die [25]). Overall, 70 mood disorder patients with suicidal behavior were divided into two groups: 34 mood disorders with SI (mean age: 30.647 ± 10.141 years; 29 females) and 36 mood disorders with SAs (mean age: 24.583 ± 8.188 years; 26 females).

### MRI acquisition

Scanning took place on a 3 T MRI scanner (General Electric, Milwaukee, USA) at the Image Institute of the First Affiliated Hospital of China Medical University, Shenyang, China. Earplugs and foam pads were used to minimize scanner noise and head motion. A standard head coil was used for radio frequency transmission and reception of the nuclear magnetic resonance signal. Three-dimensional, high- resolution, T1-weighted images were collected using a 3-D fast spoiled gradient-echo (FSPGR) sequence with the following parameters: TR/TE = 7.1/3.2 ms, image matrix = 240 × 240, field of view (FOV) = 240 × 240 mm^2^, 176 contiguous slices of 1 mm without gap, voxel size = 1.0 mm^3^. Participants were instructed to close their eyes, remain awake, and keep their mind blank during the resting state scan (after scanning we checked this with the subjects).

### Data processing

Processing was performed using the DARTEL algorithm Statistical Parametric Mapping software (SPM8, http://www.fil.ion.ucl.ac.uk/spm/software/spm8/) under the MATLAB R2010b platform (Mathworks, Sherborn, MA, USA). Segmentation function was used to divide the regions into gray matter (GM), white matter (WM), and cerebrospinal fluid (CSF) using the ‘new segment’ tool implemented in SPM8. During spatial normalization, inter-subject registration was achieved using respective registration based on group assignment. A modulation step was used to ensure that the overall amount of tissue in a class was unaltered. The segmented images were normalized to the Montreal Neurological Institute (MNI) template and were smoothed with an 8-mm full width at half-maximum (FWHM) Gaussian filter. The voxel size of data acquisition was 1 mm^3^ and the voxel size of normalized data was 1.5 mm^3^.

### Statistical analyses

Three-group (mood disorders with suicidal behavior, MC, and HC) analyses of GM volumes were performed in SPM8 using ANCOVA with a diagnostic group as an independent factor and age and gender as covariates. Statistical significance was set at significant inference of *P* < 0.01 with Family Wise Error (FWE) correction. An extent threshold of 10 voxels was considered significant among the three groups. We then extracted GMV values for each cluster with significant differences for the three-group comparison and conducted pairwise two sample t-tests, corrected for multiple comparisons (*P* < 0.05, Least Significant Difference [LSD] test).

The demographic and clinical characteristics of the subjects were analyzed using IBM SPSS Statistics for Windows, Version 22.0 (Armonk, NY, USA). Student's t-tests, one-way analyses of variance, or Chi-square tests were used depending on the normality of distribution and type of data. Categorical variables were described using frequencies and proportions. Continuous variables were presented as mean ± standard deviation. Statistical significance was determined by *P* < 0.05.

## Results

### Demographics and clinical characteristics of all participants

The results of the demographic data are listed in Tables 1 and 2. There were no significant differences among the patients with mood disorders with suicidal behavior, MC, and HC groups in age (*P* = 0.822), education (*P* = 0.247), or gender (*P* = 0.179). The HAMD total scores in the patients with mood disorders and suicidal behavior were higher than those in the MC group (*P* = 0.004). The YMRS total scores in the patients with mood disorders and suicidal behavior were lower than those in the MC group (*P* = 0.002). Education (*P* = 0.255), gender (*P* = 0.183), and HAMD total scores (*P* = 0.239) demonstrated no significant differences between the patients with mood disorders and SAs and mood disorders with SI groups. The age of the mood disorder patients with SI was higher than those in the mood disorders with SAs group (*P* = 0.048). The YMRS total scores in the mood disorder patients with SI were lower than those in the mood disorders with SAs group (*P* = 0.046).

**Table 1:** Demographics and clinical characteristics of all participants

Characteristic	Mood disorders with suicidal behavior (*N* = 70)		Mood disorders control (*N* = 126)		Health control (*N* = 144)		Analysis	
	Mean	SD	Mean	SD	Mean	SD	*F/t*	*P*
Age (years)	27.529	9.618	27.141	8.318	27.833	9.498	0.196	0.822
Education (years)	12.686	2.742	13.317	3.403	13.375	2.628	1.404	0.247
HAMD total	23.471	8.214	17.04	9.855	–	–	8.356	0.004
YMRS total	2.250	4.039	3.983	6.325	–	–	10.075	0.002

	N	%	N	%	N	%	X^2^	p
Gender (female/male)	55/15		93/35		96/48		3.437	0.179

*HAMD* Hamilton Depression Scale, *YMRS* Young Manic Rating Scale, *SD* standard deviation

**Table 2:** Demographics and clinical characteristics of participants with suicidal behavior

Characteristic	Mood disorders with suicidal attempt (*N* = 36)		Mood disorders with suicidal ideation (*N* = 34)		Analysis	
	Mean	SD	Mean	SD	*F/t*	*p*
Age (years)	24.583	8.188	30.647	10.141	4.055	0.048
Education (years)	12.306	2.55	13.088	2.917	1.32	0.255
HAMD total	23.583	8.52	23.353	8.003	1.413	0.239
YMRS total	2.914	4.955	1.545	2.659	4.143	0.046

	*N*	%	*N*	%	*X*^*2*^	*p*
Gender (female/male)	29/5		26/10		1.775	0.183

*HAMD* Hamilton Depression Scale, *YMRS* Young Manic Rating Scale, *SD* standard deviation

### GMVs findings

**Fig. 1:**
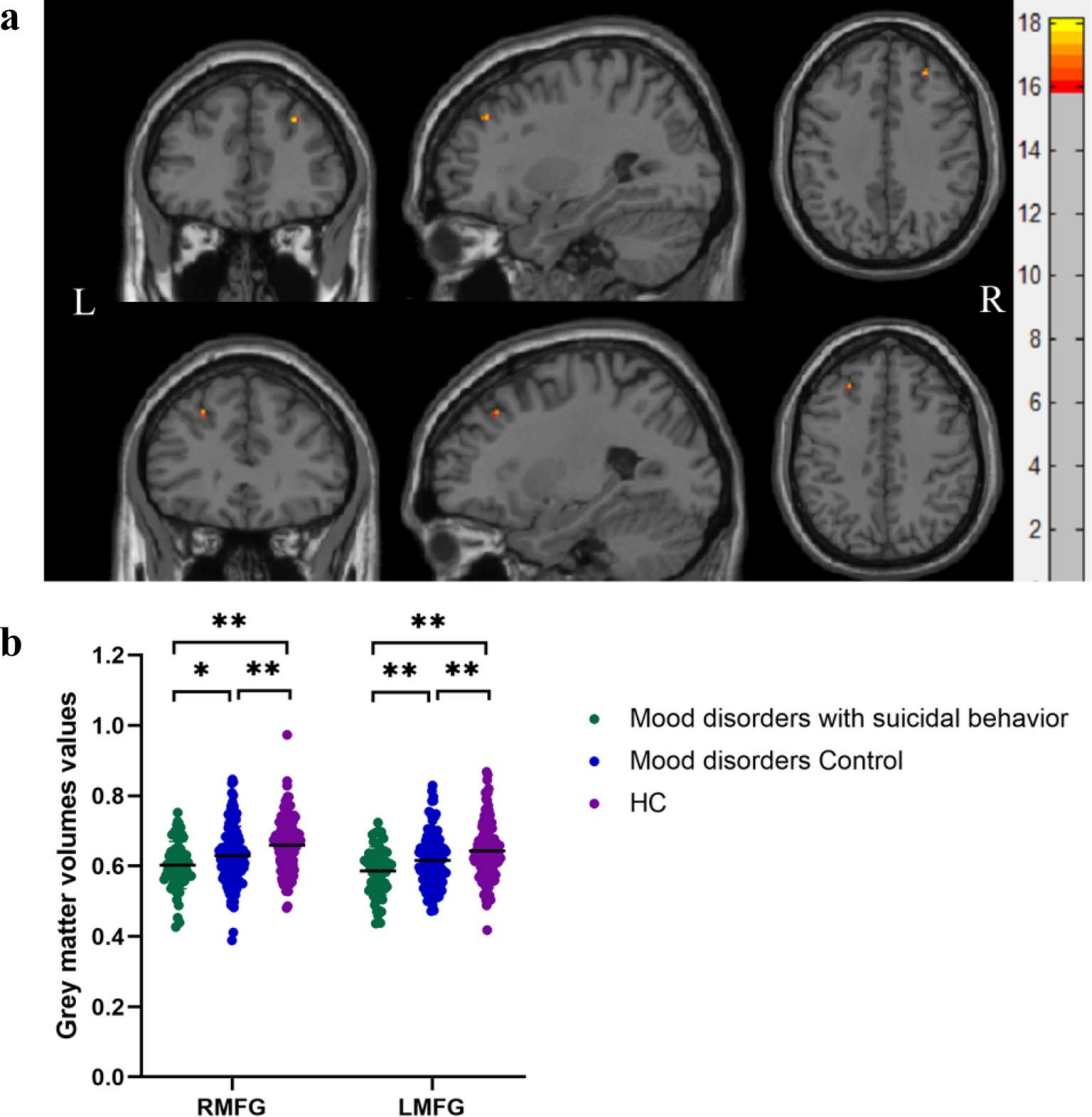
**a** Significant differences in grey matter volumes among patients with mood disorders and suicidal behavior, mood disorder controls, and healthy control groups. Significant at *P* < 0.01 corrected by Family Wise Error (FWE) correction. **b** Post hoc analysis of grey matter volumes of brain regions among mood disorders with suicidal behavior, mood disorder controls, and healthy control groups. (*P < 0.05, **P < 0.01). *RMFG* right middle frontal gyrus, *LMFG* left middle frontal gyrus

**Fig. 2:**
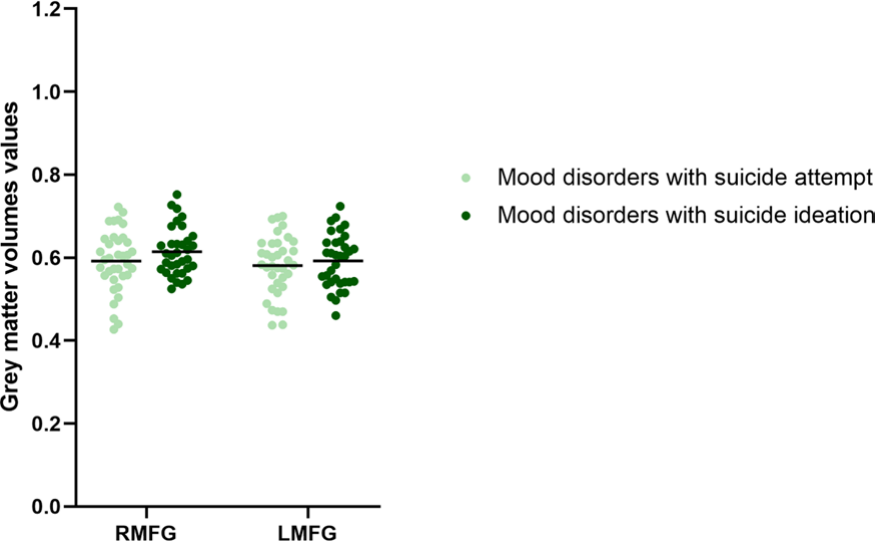
Post hoc analysis of grey matter volumes in the left and right middle frontal gyrus between patients with mood disorders and suicide attempts and mood disorders with suicidal ideation groups. *RMFG* right middle frontal gyrus, *LMFG* left middle frontal gyrus

Significant group differences were found in the GMVs of the left and right middle frontal gyrus (Table 3, Fig. 1a). Post hoc comparisons showed significant differences in the GMVs across all three groups (*P* < 0.01): HC > MC > mood disorders with suicidal behavior for the left and right middle frontal gyrus volumes (Fig. 1b). Then, we divided the mood disorder patients with suicidal behavior into mood disorders with SI and mood disorders with SAs. There were no significant differences in the GMVs of the left and right middle frontal gyrus between the patients with mood disorders and SI and mood disorders with SAs (Fig. 2). However, the average in the GMVs of the left and right middle frontal gyrus in the mood disorder patients with SAs group was lower than those in the mood disorders with SI group.

**Table 3:** Brain regions showing significant differences in grey matter volumes among mood disorders with suicidal behavior, mood disorder controls, and healthy control groups

Region	Voxel	MNI coordinates			*F* values*
		*x*	*y*	*z*	
Right middle frontal gyrus	16	28.5	39	37.5	13.622
Left middle frontal gyrus	18	− 25.5	31.5	42	13.687

*MNI* Montreal Neurological Institute. ^*^Significant at *P* < 0.01 corrected by Family Wise Error (FWE) correction

## Discussion

Our findings showed that patients with mood disorders and suicidal behavior had decreased GMVs in the right and left middle frontal gyrus, as compared to both the mood disorder control and HC groups. These results implicate the involvement of GMV alterations in the right and left middle frontal gyrus in suicidal behavior in mood disorders. The average GMVs in the right and left middle frontal gyrus in the patients with mood disorders and SAs were lower than those in the mood disorders with SI group. However, there were no significantly statistical differences in the GMVs of the right and left middle frontal gyrus between the mood disorder patients with SI and the mood disorders with SAs groups. One fMRI study found that adolescent SA youth showed significantly lower activity in related regions compared to adolescent SI youth [26], which is not consistent with our results. The inconsistency may be related to differences in methods and patient characteristics. Whether GMV alterations in the patients with mood disorders and SI are different from those in mood disorder patients with SAs needs further study. Meanwhile, the present study reported that hyposensitivity or hypersensitivity may be "trait" markers of individuals with mood disorders and interventions should refer to the individual unique sensory profiles and their behavioral and functional impact in the context of real life [27]. Thus our next work also needs to explore the relationship between above trait and suicidal behavior in mood disorders.

We observed reductions of GMV in the middle frontal gyrus of patients with mood disorders and suicidal behavior, which supports that alterations in the middle frontal gyrus may be involved in suicidal behavior. Previous studies have also supported the middle frontal gyrus being involved in suicidal behavior in mood disorders, and are consistent with our findings. For example, functional MRI studies showed that patients with MDD and SAs had abnormal brain activity in the middle frontal gyrus when compared to individuals without SAs [26, 28–30]. Additionally, patients with MDD and SI also showed a distinct brain network characterized by functional connectivity differences in the middle frontal gyrus versus the MDD patients without SI [31, 32]. Structural MRI studies showed a gray matter volume reduction in the middle frontal gyrus of patients with MDD and a history of SAs [33]. For BD patients, the predicted values of probability for attempting suicide showed a significant positive correlation with GMVs in the middle frontal gyrus [34]. With regard to mood disorders, the middle frontal gyrus had a significant association of neural activity during goal-representation with past SI and behavior in patients with mood disorders [35].

In addition, schizophrenia patients with a history of suicide attempts showed significant volumetric associations with the left middle frontal gyrus [36]. The acquired capability for suicide networks in males consisted of the middle frontal gyrus [37]. A pilot study of differential brain activation to suicidal means and DNA methylation of the CACNA1C gene in SA patients, found that the left middle frontal gyrus was shown to have significantly higher brain activation in the SA patients than the controls [38]. The SA patients with no diagnosable psychiatric disorder group exhibited significantly abnormal regional homogeneity in the middle frontal gyri when compared with a HC group [39]. The SI group showed diminished cortical volume in the left middle frontal gyrus as compared to the HC [40]. The above evidence also proves that the middle frontal gyrus may have an important correlation with suicidal behavior, and its change may be an important biological marker of suicidal behavior.

The sample size of this study is large enough. However, there are several limitations. First, we did not collect other information on duration of medication, because some patients did not precisely remember the duration of medication due to a longer duration of illness, which may have affected the results accuracy. Second, we did not collect additional information to assess the severity of the suicidal ideation and suicide attempts (e.g., Suicide Intent Scale and Columbia Suicide Severity Rating Scale), so we do not know whether our findings are related to the severity of SI or SAs. Third, we did not examine the impact of alexithymia on suicide, which will be further discussed in future studies. Meanwhile, we also did not collect the data on patients with a recent suicide attempt or those with a not recent suicide attempt, so we did not definitively differentiate information between these two groups. Finally, we collected our first data in 2012, using the DSM-IV diagnostic criteria. To ensure data consistency, in the later stage of study we did not use DSM5. We need further studies to address these limitations to better understand the complex relationship between brain structure and suicidal behavior in mood disorders.

## Conclusions

Patients with mood disorders and suicidal behavior exhibit decreased GMVs in the left and right middle frontal gyrus. However, these differences were not found between the mood disorders with SI and mood disorders with SA groups. These findings indicate that abnormal regional GMVs may be related to suicidal behavior in mood disorders. Further investigations are warranted to determine if regional GMVs could serve as a biomarker of suicidality in mood disorders. We also need further study to explore whether GMV alterations in mood disorders with SI are different from those in mood disorders with SAs.

## Data Availability

The datasets used and/or analysed during the current study are available from the corresponding author on reasonable request.
